# Motivations and attitudes towards consulting eWOM when booking accommodation

**DOI:** 10.1007/s11628-023-00533-z

**Published:** 2023-05-03

**Authors:** Beatriz Moliner-Velázquez, María Fuentes-Blasco, Irene Gil-Saura

**Affiliations:** 1 Market Research Department, Faculty of Economy, University of Valencia, Avda. de los Naranjos, s/n, 46022 Valencia, Spain; 2 Department of Management and Marketing, Faculty of Business, Pablo de Olavide University, Ctra. de Utrera, Km., 1, 41013 Seville, Spain

**Keywords:** Electronic word of mouth, Attitude, Motivations, Credibility, Hotels

## Abstract

This study explores the process of formation of attitudes towards eWOM by analyzing the relationships among “perceived credibility, volume and confirmation of consulted eWOM—motivations—attitudes towards consulted eWOM”. Using a sample of 393 Spanish hotel guests, the results confirm this chain of relationships, except for the effect of volume on motivations, and the mediating role of these motivations. The novelty of this work lies in the study of eWOM behavior from the receiver’s perspective, since it analyzes the antecedents of eWOM motivations and their relationship with the attitudes towards that information.

## Introduction

According to Internet World Stats (2022), the world Internet penetration rate is 66.2%, 88.4% in Europe and 92.5% in Spain. The Internet makes it easier for consumers to generate and disseminate information and has become an essential and unavoidable source of promotion for brands. Thanks to online media, consumers form part of product and service marketing messages (Line et al. 2020). This form of promotion, based on electronic word-of-mouth communication (hereinafter eWOM) has evolved as the generations of Internet services have changed, from Web 2.0 at the beginning of the twenty-first century, passing through Web 3.0 in 2006, to the current version of Web 4.0 (Verma and Neha Yadav 2021).

The power that a consumer’s eWOM behavior exerts on the decisions of others makes it one of the most important marketing tools in a company’s communication strategy. The literature acknowledges that online reviews are an important source of information and means of sharing opinions (Toubes et al. 2021) and there is consensus on the effect that interactions between consumers have on attitudes towards brands and purchase decisions (Brodie et al. 2013; Abubakar et al. 2016; Moliner-Velázquez et al. 2022).

The most recent literature highlights the impact of eWOM behavior on the various stages and touchpoints of the customer’s overall experience or “consumer journey” (Ngarmwongnoi et al. 2020). For this reason, there is growing academic and practical interest in the phenomenon (Yusuf et al. 2018; Zhang et al. 2021), especially in the context of tourism services (Casado-Díaz et al. 2017; Litvin et al. 2018; Tan and Lin 2021; Berné-Manero et al. 2023; Kapes et al. 2022).

There are two lines of research on eWOM behavior that respond to the focus of its study: receiver and sender (Gvili and Levy 2018; Kanje et al. 2020). Approaching eWOM from the receiver’s viewpoint consists of analyzing the search for pre-purchase information that helps the consumer to decide, while the study of eWOM from the sender’s viewpoint focuses on the post-purchase posting of comments to help others, express dissatisfaction, or reduce dissonance (Laughlin and MacDonald 2010).

The review of the literature on eWOM contains a number of gaps that are addressed in this work. Firstly, most of the works focus on the sender (Fine et al. 2017; Line et al. 2020; Shen et al. 2021; Tan and Lin 2021; Salagrama et al. 2021), and there are even fewer studies that deal with eWOM queries (Kim et al. 2011; Book and Tanford 2019; De Lima et al. 2020). However, the study of these queries in the tourism context is especially important not only before the purchase, but also during the experience itself, since browsing a hotel’s website, searching for information online or consulting forums and social networks has become a common practice for decision-making and for the service consumption phase.

Secondly, receiver-based research is primarily focused on the effects of eWOM queries on company results such as sales (Park and Lee 2009) or performance (Kim et al. 2011; Berné-Manero et al. 2020), and on aspects of consumer behavior (Ladhari and Michaud 2015), such as purchase decision (Parikh et al. 2014), purchase intention (Casado-Díaz et al. 2017; Jalilvand and Heidari 2017; Abedi et al. 2020; Anubha and Shome 2021), or purchase attitudes (Mahpour et al. 2018; Abubakar et al. 2017). However, little attention has been paid to the study of the variables that influence these queries (Hatzithomas et al. 2016). Along these variables, attitudes towards eWOM take on a fundamental role in explaining consultation behavior (Gvily and Levy 2016, 2018).

In view of these gaps, we believe that the literature should advance in the study of the origin of eWOM consultation behavior, since a better knowledge of its background will allow us to extend the study of the social influence that consumers receive when making a decision (Kim et al. 2011). The study of social influence is a topic of recent interest and concern to eWOM behavioral researchers (Yusuf et al. 2018; Kanje et al. 2020). Following this line, it is necessary to investigate the factors that condition eWOM consultations, since the empirical evidence found has been more focused on the effect of these consultations on purchases (e.g. Abedi et al. 2020; Moliner-Velázquez et al. 2022). In this issue, the study of attitudes is key to explaining behaviors, and the expectancy value theory (EVT) of Fishbein and Ajzen (1975) is the fundamental theoretical support in which the contexts linked to interactions between consumers, such as eWOM behavior, are framed (Gvili and Levy 2018).

Building on this study approach, in the context of eWOM consultations, some authors have analyzed how attitudes influence intentions (e.g. Kudeshia and Kumar 2017), but do not address the formation of attitudes toward eWOM consultations. The literature review suggests that motivations may be important antecedents. Such motivations have mainly been studied in a bid to know more about their relationship with the adoption of eWOM or the purchase decision (Parikh et al. 2014; Book and Tanford 2019; Moliner-Velázquez et al. 2022). However, no research has been found that addresses the antecedents of motivations to consult eWOM and their relationship with attitudes towards eWOM.

In order to fill the gap in the literature on the antecedents of eWOM consultations and, in particular, attitudes toward such consultations, the aim of this work is to study the process that leads to the formation of attitudes towards eWOM by analyzing the effect of the motivations for consultation and their antecedents. The antecedents proposed to explain the motivations are the eWOM attributes related to credibility and the volume of eWOM consulted (Matute-Vallejo et al. 2015), as well as the confirmation of the eWOM (Nam et al. 2020). Based on these variables, we set two objectives: (1) to analyze the chain of relationships “credibility, volume and confirmation of eWOM—motivations for consultation—attitudes towards eWOM”, and (2) to study the mediating effect that the motivations have in the relationship between their antecedents and the attitudes.

The novelty of this work lies in the study of the antecedents of consumers’ motivations to consult eWOM and their relationship with the attitudes towards that information. Although the literature offers indications that the proposed antecedents of motivations—credibility and volume—influence consumers’ attitudes towards using eWOM as an information tool for purchase decision (e.g. Yusuf et al. 2018); Bulut and Karabulut 2018), no evidence has been found on their relationship with motivations. That is why this paper contributes by advancing this line of research and providing empirical evidence on the relationships between variables influencing attitudes towards eWOM consultations. Therefore, the results of this research extend the study of eWOM behavior from the receiver’s perspective and the factors that influence eWOM consultations, a field that has been scarcely investigated compared to research on eWOM dissemination after the experience. In particular, we contribute to the study of attitudes towards the consulted eWOM, as a key element that conditions the intentions towards its use and the consultation behavior (Fishbein and Ajzen 1975; Ajzen 1991).

The remainder of this paper is structured as follows. Sections 2 and 3 provide the literature review and develop the hypothesis according to the proposed model. Section 4 describes the research methodology and Sect. 5 explains the results of the data analysis. Finally, Sect. 6 presents the conclusions and includes implications and future research.

## Theoretical review

eWOM behavior can be defined as any positive or negative statement about a product/service/company/brand posted on the Internet by current, potential, or former consumers (Hennig-Thurau et al. 2004). The review of the literature shows some focuses of debate that pose significant challenges to the advancement of research. These focuses mainly refer to four areas of study: the role adopted by the consumer (sender and/or receiver), the information content (recommendation, comment, etc.), the differences with respect to traditional WOM, and the measurable dimensions (credibility, valence, emotions, etc.).

From the receiver approach, the study of eWOM follows the process by which an individual receives and applies information, based on the Theory of Reasoned Action (TRA) (Fishbein and Ajzen 1975) and the Theory of Planned Behavior (TPB) (Ajzen 1991). This process is known as information adoption and is defined as a psychological action that affects consumers when they are exposed to social norms, reviews, or comments in the online medium (Fan et al. 2013). The theoretical models that support this process are the Technology Acceptance Model (TAM) (Davis 1989), the Information Acceptance Model (IAM) (Sussman and Siegal 2003) and the extended IAM model (IACM) by Erkan and Evans (2016), which have been used in several works on eWOM (Cheung et al. 2008; Rahman and Mannan 2018; Le-Hoang 2020).

According to the Elaboration Likelihood Model (ELM) (Petty and Cacioppo 1986), consumers are affected by the information they receive either via the central route or the peripheral route (Sussman and Siegal 2003). The central route is the decision made by an engaged consumer based on rational criteria, such as the quality of the information received. However, the peripheral route is more typical of a consumer who is less engaged, as they are more likely to be influenced by aspects that require less cognitive effort, such as the image of the product in question, the quantity and/or the credibility of the information.

The effect of the eWOM queries that a consumer makes on their decisions is especially important and even surpasses the effect exerted not only by other forms of promotion or communication, but also their own experience (Steffes and Burgee 2009). Numerous investigations confirm the influence of eWOM queries on purchase decisions (Abubakar et al. 2016; Casado-Díaz et al. 2017; Abedi et al. 2020). However, attitudes towards these consultations have not been fully addressed. Following the TRA (Fishbein and Ajzen 1975) and TPB (Ajzen 1991) models, attitudes play a leading role in eWOM, since they precede the intentions that lead to this consultation behavior. Several works have used these theories to address the relationships between eWOM information and purchase intentions, but not in regard to attitudes (Reichelt et al. 2014; Erkan and Evans 2016).

Attitudes towards eWOM play a crucial role in explaining comment query behavior. Attitude can be defined as a positive or negative psychological bias that arises from the evaluation of an object (Eagly and Chaiken 1993). The antecedents and effects of attitudes have been widely researched in marketing. In the context of communications between consumers, attitude towards eWOM behavior is a key antecedent of the response provided to the message transmitted in that communication (Gvili and Levy 2018). The Expectancy Value Theory (EVT) of Fishbein and Ajzen (1975) supports the formation of consumer attitudes towards eWOM behavior. In fact, this theoretical framework has been used in various marketing contexts, including explanations of traditional WOM behavior (Smith and Vogt 1995). According to this theory, an individual’s evaluation of an object determines their attitudes; attitudes influence intentions, and, in turn, intentions influence behavior.

In addition to attitudes, consumer motivations are also a key factor when delving into eWOM query behavior. The literature in this regard is not abundant and shows a marked lack of consensus. Various works can be found that provide classifications or propose types of motivations that can influence consultations (Parikh et al. 2014; Srivastava and Kalro 2018). One of the most widely recognized is that of Kim et al. (2011), who classify motivations into three groups: convenience, risk reduction, and social.

Convenience motivations focus on reducing the effort and costs of searching for information. These can be internal costs, when they refer to the mental effort of searching, classifying, integrating, and interpreting the information found, and external costs, when it comes to the monetary expenditure and search time (Goldsmith and Horowitz 2006). Consumers motivated by these types of factors seek to streamline search tasks and time, find unbiased information to reduce cognitive dissonance, and obtain greater value for money (Hennig-Thurau and Walsh 2003).

Risk reduction motivations seek to reduce four types of risk: functional risk, related to the benefits perceived before the purchase; social risk, related to the acceptance and recognition of others; economic risk; and physical risk (Flanagin et al. 2014). This type of motivation allows consumers who consult eWOM to better predict their purchases and reduce the probability of failure (Parikh et al. 2014). This is especially important for services, where consumers often seek information to reduce their intangibility, inseparability, variability, and expiration before deciding to purchase.

Finally, social motivations focus on the search for security, approval, and belonging to a group (Abrantes et al. 2013). Social groups, and reference groups in particular, have been recognized in the literature as motivating elements of eWOM searches due to the pressure and conformity effect they exert on the individual (Parikh et al. 2014). Additionally, consumers who act based on these reasons are looking to satisfy the need for curiosity, knowledge, and learning about products and services (Hennig-Thurau and Walsh 2003).

## Proposed model and hypothesis

### Antecedents of the motivations

One of the advantages of eWOM communication is that it is widely accessible through various online media, which facilitates interaction between consumers who are unknown to each other. In fact, some studies highlight that the anonymity offered by the Internet can raise doubts about the veracity of published comments (Luo et al. 2013). For this reason, certain characteristics or attributes of eWOM acquire special importance. The literature on eWOM from the receiving consumer approach suggests that some characteristics of this behavior, credibility and the amount of information in particular, can determine attitudes (Zhu and Zhang 2010; Park et al. 2011). There is greater consensus about the nature and dimensionality of these particular eWOM attributes (Matute-Vallejo et al. 2015). Furthermore, in line with the IAM model (Petty and Cacioppo 1986), both attributes are considered determining factors of the peripheral route, since they especially affect consumers who are not very engaged or are less capable of processing and understanding information (Park and Kim 2008).

The credibility of the information that is transmitted through eWOM is based on the beliefs formed by the individual from reading comments posted by other consumers on the Internet (Cheung et al. 2008). It can be defined as the degree to which a consumer perceives that the recommendations of others are credible and truthful. This characteristic of eWOM generates a certain degree of confidence in the consumer since it is based on objective data that comes from customer experiences and is not commercially motivated. This facilitates the communication process (Matute-Vallejo et al. 2016), in which social networks have become essential platforms because they favor and support relationships not only between consumers but also between consumers and brands. That is why companies prefer to launch messages about their brands through these media that consumers value and perceive as credible sources (Gvili and Levy 2018).

The literature indicates that credibility plays a key role in the eWOM communication process as it reinforces the consumer’s attitude and increases purchase intentions (Matute-Vallejo et al. 2016; Bi and Zhang 2022). The work of Rahman and Mannan (2018) confirms that credibility mediates the relationship between the adoption of eWOM information and the online purchase decision. Some studies have confirmed the effect that credibility has on attitude. The work of Gvili and Levy (2018) shows that the perceived credibility of eWOM positively influences the consumer’s attitude towards eWOM in social networks. Also, Yusuf et al. (2018) find that credibility exerts a positive effect on commitment to the use of eWOM. When consumers judge eWOM information to be credible, they are more willing to engage in this form of communication (Cheung and Thadani 2012). According to their extended IAM model (IACM), Erkan and Evans (2016) argue that the perception of credibility helps consumers to form attitudes towards eWOM as they consider the information to be useful and relevant, attitudes that will influence adoption of the information consulted and purchase intention. This approach has been taken by Abedi et al. (2020) in their recent work, in which it is confirmed that the credibility of the eWOM positively influences the usefulness of that information, and, in turn, this usefulness favors positive attitudes.

**Fig. 1:**
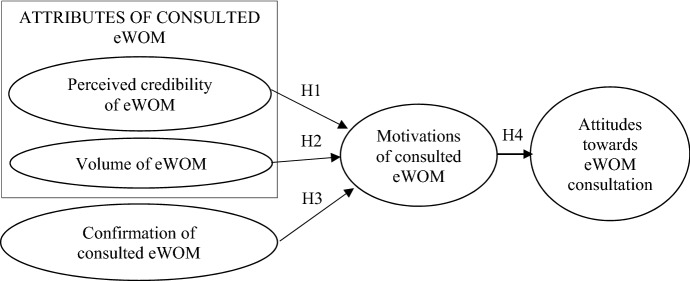
Research model

According to these results, we understand that credibility is a motivating factor that contributes to the formation of attitudes. The consumer’s perception of the veracity of the comments generates confidence and security, thereby reducing decision risk and the probability of failure (Parikh et al. 2014). Therefore, we assume that credibility will have a positive effect on the motivations leading up to the eWOM query (Fig. 1):

#### H1

The perceived credibility of eWOM positively influences the motivations to consult eWOM.

In general, the volume of eWOM refers to the number of comments and evaluations about products made by consumers through online media (Park et al. 2011; Zhu and Zhang 2010; Matute-Vallejo et al. 2015). The literature also addresses the concept of quantity of eWOM information as the number of comments shared via a web page (Sicilia and Ruiz 2010). Some contributions consider that it may indicate the degree of risk involved in a purchase decision (Bond et al. 2019). A high number of ratings can increase confidence (Hu and Kim 2018) and the perception of usefulness of the eWOM (Park and Lee 2009). Likewise, volume is considered to be an indicator of the popularity of a product because it reflects the interest of the market (Moldovan et al. 2011).

Different investigations highlight the effects that the volume of eWOM has on aspects such as the rationalization of the purchase decision and the reduction of perceived risk. Along these lines, a high volume of comments indicates that there is more information available on which to base a decision (Bond et al. 2019) and this gives the consumer peace of mind when making it (Ngarmwongnoi et al. 2020). It has also been highlighted that volume favors image and confidence in the company (Sparks and Browning 2011), generates a favorable attitude, and is related to purchase intentions (Bataineh 2015; Bulut and Karabulut 2018). Considering that a large part of the motivations that the consumer has to consult eWOM seeks to reduce the effort, costs, and risk of the decision, it is expected that the volume of eWOM available for consultation will motivate the individual to take comments into consideration when making their decision (Fig. 1):

#### H2

The volume of eWOM positively influences the motivations to consult eWOM.

Consumers making a purchase decision may have been swayed by eWOM comments because they find them to be sufficient in number and credible. However, it is also important for the consumer to verify whether these comments are proven to be accurate, as this will condition the use of eWOM queries for future decisions. Therefore, in addition to the credibility and volume of eWOM, it is suggested that the degree of confirmation of the consulted eWOM may also be a motivating factor for this behavior.

Based on the Expectation Confirmation Model (ECM) (Bhattacherjee 2001), in the context of eWOM queries, consumers form expectations about their experience based on the comments consulted, which will be confirmed or not. Therefore, the confirmation of these expectations is predicted to be related to the behavior of continuing to consult eWOM. Along these lines, in the work of Nam et al. (2020) it is verified that lack of confirmation in regard to the eWOM consulted in previous experiences is positively related to distrust in those comments. We understand that confirmation of the eWOM that the consumer has previously consulted will generate confidence when consulting eWOM, thus reducing the perception of risk in their decision. Therefore, it is a factor that can influence the consumer’s motivations to consult eWOM (Fig. 1):

#### H3

Confirmation of the consulted eWOM positively influences the motivations to consult eWOM.

### Effect of motivations on attitude

The few works that address attitudes towards eWOM analyze its effects on commitment (Yusuf et al. 2018; Gvili and Levy 2018), on purchase intentions (Yusuf et al. 2018; Abedi et al. 2020), or on information adoption (Ngarmwongnoi et al. 2020). There are also limited studies on eWOM that analyze the antecedents of attitudes, including perceived credibility. For example, Abedi et al. (2020) confirm that attitudes towards eWOM information depend on the usefulness of this information and also have a direct and positive effect on purchase intention.

However, no contributions have been found that analyze the relationship between motivations to consult eWOM and attitudes. From the sender’s approach, some research has addressed the effect of motivations on attitudes towards the dissemination of eWOM (Fu et al. 2015). In the context of the receiver, certain authors analyze the influence that different types of motivations have on the intention to consult eWOM, but not on attitudes (Farzin and Fattahi 2018).

Consequently, there is limited empirical evidence directly or indirectly related to the effect that motivations have on attitudes. According to TRA (Fishbein and Ajzen 1975) and TPB (Ajzen 1991), it is important to explain the attitudes that underlie the intentions of the subsequent behaviors, whether they are eWOM consultation behaviors or purchase behaviors. If there is evidence from the sender’s perspective that the motivations to engage with eWOM have a significant effect on attitudes towards eWOM (Fu et al. 2015), we understand that this effect may also exist when we refer to the eWOM receiver. That is, we assume that consumers who feel motivated to consult eWOM will have more positive attitudes towards eWOM (Fig. 1):

#### H4

The motivations to consult eWOM positively influence the attitude towards consulting eWOM.

## Methodology

### Measurement scales and fieldwork

Quantitative research was carried out by means of a questionnaire conducted with hotel guests following the reopening of the accommodation sector in the recovery period after the first lockdown of the pandemic. The survey brought together a set of scales adapted from the literature that had previously been validated (Table 1). The measure used is a 7-point Likert scale (from 1 = strongly disagree to 7 = strongly agree). Once the scales for measuring the variables of interest had been established, a questionnaire was drafted in English, and then translated into Spanish. The questionnaire was then translated back into English to ensure that there were no inconsistencies between the two versions. In addition, a pilot test was conducted among five academic researchers with expertise in electronic consumer behavior to verify that there were no inconsistencies in the expression of each statement. Subsequently, based on the recommendations of Douglas and Craig (2007), a pilot survey was carried out on ten guests to check if the respondents understood the items, which verified that there was no problem of complexity related to the grammatical and linguistic structure or the cognitive response process (Menold 2020). After this phase, the final version of the questionnaire was prepared.

**Table 1:** Measurement model

Scale	Statement	SL (*t*-Stat)	CR	AVE
Perceived credibility of eWOM (adapted from Matute-Vallejo et al. 2015)	In general, I think the reviews and ratings of my favorite social media are credible	*0.916*	0.937	0.833
	I think I can trust the reviews of my favorite social media	0.944** (32.09)		
	I think the comments and opinions published on my favorite social media tell the truth	0.877** (22.99)		
Volume of eWOM (adapted from Matute-Vallejo et al. 2015)	My favorite social media offers many recommendations and reviews from other consumers	*0.931*	0.894	0.808
	A large number of consumers write their comments and ratings on my favorite social media	0.866** (20.08)		
Confirmation of consulted eWOM (adapted from Nam et al. 2020)	My experience with the hotel was as good as previous reviews described on social media	*0.926*	0.952	0.870
	The service provided by the hotel was as good as previous reviews described on social media	0.952** (45.38)		
	Overall, most of my expectations with the hotel were met as per previous reviews described on social media	0.920** (34.28)		
Motivations to consult eWOM (adapted from Kim et al. 2011)	I read information from other consumers’ online reviews			
	– Because it’s the fastest way to get information	*0.888*	0.953	0.719
	– Because it’s convenient to search from home or work	0.900** (25.42)		
	– Because I am able to compare different products or services easily	0.900** (30.93)		
	– To find solutions for my problems regarding shopping or consumption	0.880** (32.31)		
	– Because customer reviews help me to make the right buying decisions	0.890** (29.57)		
	– To benefit from others’ experiences when making a decision	0.895** (28.54)		
	– Because I like being part of a community	0.645** (17.05)		
	– Because I get to know about the most current topics	0.746** (20.35)		
Attitudes towards the eWOM consultation (adapted from Gvili and Levy 2018)	I like to read other people’s comments on social media about shopping or consumption experiences that interest me	*0.855*	0.857	0.751
	In general, consulting other people’s comments on social media is a good and positive experience	0.885** (21.56)		

*SL* standardized loading; *CR* composite reliability; *AVE* average variance extracted

**p < 0.01

In italics, factor loading is fixed to 1 (variance of the latent construct is free)

The fieldwork was conducted through a panel of national guests, applying quotas by age and autonomous community where the hotel was located. It was conducted by a specialist company during October 2020, the period when accommodation reopened following the enforced isolation period during COVID-19. As a result of the fieldwork, 393 valid and complete questionnaires were obtained.

The main characteristics of the sample were as follows (Table 2): 50.9% are women, with a mean age of 43.8 years (± 15 years), and 10.7% of the respondents are over 65 years of age. 62.6% are employed workers, and more than half of the sample has completed at least university level studies (60.8%). Regarding the characteristics of the experience, 93.9% indicated that the reason for the trip was leisure or vacation; 47.8% made the reservation through an online travel agency and 39.4% directly through the hotel website. 30.3% made this reservation less than a week in advance. Finally, 58% booked accommodation in a four-star hotel, and 6.9% in one of five stars or more.

**Table 2:** Sample profile

Gender		Purpose of the trip	
Male	49.1%	Leisure/Vacations	93.9%
Female	50.9%	Business	5.6%
Age		Others	0.5%
18–25 years	14.5%	Hotel	
26–35 years	19.6%	Two-star	2.8%
36–45 years	21.4%	Three-star	32.3%
46–55 years	19.3%	Four-star	58.0%
56–65 years	14.5%	Five-star or more	6.9%
> 65 years	10.7%	Advance time for reservation	
Occupation		< 1 week	30.3%
Student	7.1%	1 week to < 1 month	48.9%
Employee	62.6%	≥ 1 month to < 3 months	13.7%
Self-employed	8.9%	≥ 3 months	7.1%
Unemployed	6.9%	Stay days	
Homemaker	2.0%	≤ 2 days	31.8%
Retired	12.5%	≥ 3 days	68.2%
Education level		Reservation through	
Elementary	6.6%	Physical travel agency	6.4%
Secondary/Collage	14.0%	Online travel agency	47.8%
Vocational training	18.6%	Hotel website	39.4%
University	54.7%	Others	6.4%
Postgraduate level	6.1%		

### Reliability and validity of scales

A first-order measurement model with Robust ML was estimated to assess the dimensionality, reliability, and validity of the scales used. The indices obtained (*χ*^2^_Sat-B_./*df* = 370.89/125 = 2.97, RMSEA = 0.071, CFI = 0.943, IFI = 0.944, GFI = 0.860, BB-NFI = 0.917, BB-NNFI = 0.931) indicate that the model shows a proper fit. All the constructs were unidimensional, considering the dimensions of convenience, risk reduction, and social motivations separately for the eWOM consultation.

Internal consistency was evaluated through the composite reliability coefficient (CR > 0.7) and the average variance extracted (AVE > 0.5), exceeding the minimum thresholds defined by Anderson and Gerbing (1988) and Fornell and Larcker (1981). These values, along with the standard loadings, are shown in Table 3.

**Table 3:** Descriptive statistics and measurement scales correlations

	Mean	SD	1	2	3	4	5
1. Perceived credibility of eWOM	4.65	1.43	*0.913*				
2. Volume of eWOM	4.42	1.64	0.683	*0.899*			
3. Confirmation of consulted eWOM	5.17	1.44	0.579	0.571	*0.933*		
4. Motivations to consult eWOM	5.01	1.39	0.629	0.529	0.633	*0.848*	
5. Attitudes towards the eWOM consultation	4.74	1.53	0.618	0.506	0.624	0.805	*0.866*

Diagonal elements (italics) are the square root of AVE

*SD* standard deviation

In relation to the validity of the measurement scales, the proposals used are endowed with convergent validity since all the factor loadings associated with the items were significant at 99% (*t*-statistic > 2.58). We can also confirm that the scales have discriminant validity, since, according to the Fornell and Larcker’s (1981) criterion, the linear correlation between each pair of latent factors is less than the square root of the factors involved (Table 3) and the differences test Δ*χ*^2^ = 101.96 (*df* = 10) is significant at 99% (Anderson and Gerbing 1988).

### Common method bias test

As indicated by Min et al. (2016), it is necessary to pay special attention to the possible common method bias (CMB) in hospitality research. To do this, following Malhotra et al. (2006), Harman’s single factor test was conducted, checking whether the fit of the measurement model from Fig. 1 was significantly better than the fit where all the observable items loaded on a single factor. The fit indices of the latter, more restricted model (*χ*^2^_Sat-Bt_/*df* = 1671.44/135 = 12.38, RMSEA = 0.170, CFI = 0.645, IFI = 0.647, GFI = 0.539, BB-NFI = 0.627, BB-NNFI = 0.598) were significantly worse than those of the measurement model shown in the previous section at the 0.01 level (Δ*χ*^2^(*df* = 10) = 1360.20; *p* < 0.000). Furthermore, the correlations between the latent constructs were significant and less than 0.9 (Bagozzi et al. 1991).

## Results

A causal model was estimated that encompassed the relationships presented in Fig. 1. The indices obtained allow us to consider an estimate with adequate adjustment (*χ*^2^_Sat.-Bt_/*df* = 376.32/128 = 2.94, RMSEA = 0.070, CFI = 0.943, IFI = 0.943, GFI = 0.859, BB-NFI = 0.916, BB-NNFI = 0.931). Based on the standardized coefficients shown in Table 4, the results indicate that there is a positive and significant effect of the perceived credibility attribute of the eWOM on the motivations for consultation, thereby confirming hypothesis H1. However, there is no significant effect of the other eWOM attribute, the volume, on the motivations to consult, and therefore it is not possible to validate hypothesis H2. Regarding the last antecedent, we can conclude that confirmation of the consulted eWOM has a positive and considerable influence on the motivations, confirming hypothesis H3. In addition, the estimate indicates that the motivations have a significant and positive effect on attitudes towards eWOM consultation. Therefore, we can affirmatively confirm hypothesis H4. Overall, the antecedents explain that *R*^2^ = 82.5% of the variability of the attitudes. Therefore, these results respond to the first objective set by confirming the chain of relationships “perceived credibility and confirmation of consulted eWOM—motivations—attitudes towards eWOM”.

**Table 4:** Structural model estimation

H	Relationship	Standardized coefficient	*t*-test	Supported
H1	Perceived credibility of eWOM → Motivations to consult eWOM	0.369**	4.47	Yes
H2	Volume of eWOM → Motivations to consult eWOM	0.055	0.80	No
H3	Confirmation of consulted eWOM → Motivations to consult eWOM	0.393**	6.47	Yes
H4	Motivations to consult eWOM → Attitudes towards eWOM consultation	0.908**	20.35	Yes

**Significant at 0.01

Another objective of this proposal is to verify the indirect effect of both eWOM attributes (credibility and volume) and confirmation on attitudes, mediated by motivation. The results indicate that there is a significant indirect effect of perceived credibility (0.399**; *t*-Stat = 4.35) and confirmation (0.359**; *t*-Stat = 6.17) on attitudes, but not so between volume and attitudes. To confirm these effects, a more comprehensive mediation analysis of the motivations to consult eWOM was conducted, testing whether the unstated direct relationships between antecedents of motivations and attitudes were significant (Table 5).

**Table 5:** Mediation test

Model	*χ*^2^ Stat	Additional stand. coefficient	*χ*^2^ difference
M1 = Fig. 1	*χ*^2^_Sat._(*df* = 128) = 376.32 *χ*^2^(*df* = 128) = 579.07		
M2 = M1 + Perceived credibility of eWOM → Attitudes towards eWOM consultation	*χ*^2^_Sat_.(*df* = 127) = 374.10 *χ*^2^(*df* = 127) = 575.41	*γ* = 0.08 (*t*-Stat = 1.58)	*χ*^2^ (M1–M2) (*df* = 1) = 2.26 (*p*-value = 0.133007)
M3 = M1 + Confirmation of consulted eWOM → Attitudes towards eWOM consultation	*χ*^2^_Sat_.(*df* = 127) = 372.58 *χ*^2^(*df* = 127) = 574.81	*γ* = 0.09* (*t*-Stat = 1.97)	*χ*^2^ (M1–M3) (*df* = 1) = 4.14* (*p*-value = 0.041900)

*: significant at 0.05; **: significant at 0.01

The direct relationship between the perceived credibility of eWOM and the attitudes towards eWOM queries is not significant (*γ* = 0.08) (Table 5). Therefore, we can conclude that the motivations to consult eWOM exert a complete mediation between both constructs (Nitzl et al. 2016). In the case of the confirmation of the consulted eWOM, this antecedent does have a significant direct influence, at 95%, on attitudes (*γ* = 0.09*), so we can conclude that there is a partial mediation of the motivations between the confirmation and the attitudes. In conclusion, motivations to consult eWOM mediate the effect of credibility and confirmation on attitudes, this being total mediation in the case of credibility and partial mediation in the case of confirmation.

## Conclusions and further research

Regarding the first objective, to analyze the chain of relationships “credibility, volume and confirmation—motivations—attitudes towards eWOM”, we can conclude that the motivations to consult eWOM play a key role in the formation of attitudes towards this information. In particular, it is confirmed that, of the three antecedents of the proposed motivations, credibility and confirmation of the consulted eWOM positively influence the motivations to consult. When the consumer perceives that the comments read are truthful, they consider the information to be useful and relevant (Abedi et al. 2020) and this generates greater confidence in decision-making and less risk or probability of failure (Parikh et al. 2014). In addition, the way in which the consumer evaluates the eWOM queries made creates expectations that may or may not be confirmed. If the consumer feels, in their experience with the service, that these comments have been accurate, the fulfillment of these expectations will generate trust for future decisions. This result is in line with the work of Nam et al. (2020), who find a positive relationship between eWOM disconfirmation and mistrust.

These findings allow us to extend the study of the motivations for consulting eWOM, since it has been demonstrated that perceived credibility and confirmation of eWOM are important motivating factors for consultations. In particular, they are variables that increase trust, simplify information searches, reduce decision risk, and provide greater security in the purchase, so they are clearly linked to convenience, risk reduction, and social motivations (Kim et al. 2011).

No significant effects of the volume of eWOM consulted on motivations have been found. Although the literature has highlighted that the number of comments increases consumer confidence (Hu and Kim 2018), reduces the perceived risk (Park and Lee 2009), and gives peace of mind when deciding to purchase (Ngarmwongnoi et al. 2020), our results show that the volume does not influence motivations to consult eWOM. Comparable results are found in the work of Matute-Vallejo et al. (2015) who confirm that the volume of eWOM does not contribute to trust in or attitude towards the seller.

Of the two eWOM attributes proposed as antecedents of motivations (credibility and volume), only perceived credibility has been found to have a significant effect. According to the ELM model (Petty and Cacioppo 1986), credibility and volume are criteria used by consumers who receive and adopt information via the peripheral route. In our case, given that the consumer is influenced to feel motivated to make enquiries by the perception of credibility of eWOM and not by the volume, it can be concluded that the consumer partially follows the peripheral route. The profile of the sample may support this conclusion. The fact that almost 80% of the sample had booked the hotel less than 2 weeks in advance and almost 90% had made the purchase online (Table 2) could indicate that we are dealing with consumers with low levels of engagement with their decision, seeking to reduce the cognitive effort of the consultations and letting themselves be influenced more by the perception of credibility than by the quality of the information (Park and Kim 2008).

Regarding the second objective, to study the mediating effect of motivations on the relationship between their antecedents and the attitudes, the results show two interesting mediating effects. On the one hand, motivations to consult eWOM fully mediate the relationship between perceived credibility and attitudes. That is, credibility influences attitudes only through motivations. This implies that the formation of attitudes towards consulting eWOM does not depend on credibility but on the different motivations that the individual has for consulting information. Furthermore, motivations only partially mediate the relationship between eWOM confirmation and attitudes. This partial mediation implies that the confirmation of the consulted eWOM is revealed as a particularly important variable in the formation of attitudes towards consultations, since it has not only a direct influence but also indirect through motivations.

In summary, it has been revealed that attitudes towards queries depend on the consumer’s motivations and these motivations are determined by the credibility and confirmation of the eWOM consulted. These results have relevant implications for advancing the study of the antecedents of eWOM queries. We contribute to the advancement of research on eWOM behavior from the receiver’s approach, delving into attitudes towards consultations, which will condition subsequent decisions. Although there are contributions on the effects that eWOM queries have on purchase intentions or decisions (Park and Lee 2009; Parikh et al. 2014; Casado-Díaz et al. 2017; Abedi et al. 2020; Moliner-Velázquez et al. 2022), there is no known evidence on the process prior to these intentions as the literature in the context of eWOM has focused more on intentions than on attitudes (Erkan and Evans 2016). In fact, the scarce empirical evidence found is along the lines of the effects of consultations on the purchase process (e.g. Casado-Díaz et al. 2017). Therefore, unlike previous literature, this paper contributes to the study of the origin of consumer eWOM queries by delving into their attitudes towards this behavior from the motivations and their antecedents in the context of interpersonal online communications. Since attitudes are a key element in intentions and behaviors (Fishbein and Ajzen 1975; Ajzen 1991), this work shows the need for further research on the formation of attitudes towards eWOM queries and their antecedents.

From a practical perspective, this work can be helpful in the design of hotels’ online communication actions. Marketers should be aware that consumers who consult eWOM to make their reservation decision will trust reviews that appear credible and will not be influenced by the number of reviews written by other consumers. In this regard, it would be interesting to design mechanisms that make it possible to easily select those comments that come from real clients and eliminate those written by fictitious clients or by those who seek to damage the company’s reputation. In this way, consumers who make the reservation based on the consulted eWOM will be able to verify if the experience in the hotel is a true reflection of the information received. Therefore, it is essential for the hotel to continually review customer comments, not only the negative ones to improve services, but also the positive ones to ensure that the hotel meets or exceeds the expectations created by the eWOM consulted.

From a theoretical perspective, it would be interesting to extend the relationship model by considering the dimensionality of certain variables or adding constructs to improve the explanation of eWOM motivations and queries. To do this, first of all, it is possible to delve into the analysis of motivations by addressing different dimensions. Following the classification of Kim et al. (2011), differentiating the convenience, risk reduction, and social motivations would provide information on what factors influence each type of motivation and, in turn, what type of motivation contributes the most to attitudes towards eWOM consultation.

Secondly, it is proposed to add the quality of the eWOM information as an antecedent to motivations. This eWOM attribute, together with the credibility and quantity proposed in this work, constitute the three elements of eWOM that the literature highlights for their ability to influence the attitudes and behaviors of consumers (Zhu and Zhang 2010; Park et al. 2011). Therefore, analyzing the effect that these eWOM characteristics have on motivations would help to identify which antecedent exerts most influence. According to the ELM model of Petty and Cacioppo (1986), this analysis could confirm whether consumers receive information via the central route if they are more influenced by the quality of the eWOM, or the peripheral route if they are affected more by credibility and volume. Along these lines, certain works have addressed the effect that these attributes can have on client responses. For example, the works of Matute-Vallejo et al. (2015, 2016) confirm that information quality, quantity, and credibility influence the perception of usefulness. Also, Abedi et al. (2020) find that credibility and quality influence utility. However, no evidence has been found on the effect of these three eWOM characteristics on the motivations to consult, so it is of interest to address the different effects that these antecedents could have on the three groups of motivations.

Thirdly, the model proposed in this work could also be improved by advances in the chain of relationships to explain the actual eWOM consultation behavior. Taking support from the TRA and EVT theories of Fishbein and Ajzen (1975) that relate “attitude—intention—behavior”, as some contributions have done in the context of eWOM (Rahman and Mannan 2018; Le-Hoang 2020), we propose to extend the study by addressing the influence that attitudes towards eWOM consultation would have on consultation intention and behavior.

From a methodological perspective, we are also aware of some concerns of the study. Some of the item statements related to credibility and volume of eWOM scales include two stimuli (“reviews and rating”, “comments and opinions” and “recommendations and reviews”). Although they can be considered double-barreled questions, we decided to keep the original wording as it was proposed by Matute-Vallejo et al. (2015). The main reason is the current debate about the content of the eWOM message. Some authors understand that eWOM is an active recommendation (Gremler and Brown 1999), others that it is simply a comment on a product or service (Sweeney et al. 2012), others a combination of both elements (Maxham and Netemeyer 2002), and even that they are extremely positive or negative comments about lived, original and memorable experiences (Anderson 1998). As we have shown in the methodology section, two pilot tests were performed among academics and hotel guests to avoid any misunderstanding. In order to avoid potential issues arising from double-barreled questions in future work, we propose to adapt eWOM credibility and volume scales that include a single stimulus. Matute-Vallejo et al. (2016) propose the term review or recommendation, but not both terms in the same statement.

On the other hand, the analysis was performed on a representative sample of national tourists. We consider that the sample could be extended to other geographical areas to improve the extrapolation of the results. In this line, other tourist contexts could be analyzed (e.g. cruises, rentals or exchange of accommodation), to see if there are differences in the relationships analyzed depending on the type of service. Also in future works, the data collection context could be considered as a moderating factor of the relationships. Along these lines, the data for this research was collected in the year 2020, a year described as the worst in the history of tourism (UNWTO 2021) and marked by a 66% reduction in income in the Spanish hotel sector (Statista 2021). This situation brought about significant changes in consumers’ purchasing behaviors, especially in the pre-purchase stage. The restrictions and security measures imposed favored rational purchases and short notice for reservations. This has notably affected consumers’ information search process since some of their motivations for consulting eWOM could have been altered. Even the perception of credibility and volume of eWOM could also be conditioned by this uncertain context. In summary, these issues help to advance this line of research by analyzing the moderating role that contexts could have, either depending on the type of service or depending on the social setting.

## Data Availability

The dataset that supports the findings of this study is available from the corresponding author on request.
